# In Situ Nanofibrillar Polypropylene-Based Composite Microcellular Foams with Enhanced Mechanical and Flame-Retardant Performances

**DOI:** 10.3390/polym15061497

**Published:** 2023-03-17

**Authors:** Yufan Jiang, Jing Jiang, Lian Yang, Yihe Zhang, Xiaofeng Wang, Na Zhao, Jianhua Hou, Qian Li

**Affiliations:** 1 School of Materials Science & Engineering, Zhengzhou University, Zhengzhou 450001, China; 2 School of Mechanics and Safety Engineering, National Center for International Research of Micro-Nano Molding Technology, Zhengzhou University, Zhengzhou 450001, China; 3 School of Mechanical & Power Engineering, Zhengzhou University, Zhengzhou 450001, China

**Keywords:** polypropylene, in situ fibrillation, microcellular foaming, compressive property, flame retardancy

## Abstract

With the increasing demand for plastic components, the development of lightweight, high strength and functionalized polypropylene (PP) from a cost-effective and environmentally friendly process is critical for resource conservation. In situ fibrillation (INF) and supercritical CO_2_ (scCO_2_) foaming technology were combined in this work to fabricate PP foams. Polyethylene terephthalate (PET) and poly(diaryloxyphosphazene)(PDPP) particles were applied to fabricate in situ fibrillated PP/PET/PDPP composite foams with enhanced mechanical properties and favorable flame-retardant performance. The existence of PET nanofibrils with a diameter of 270 nm were uniformly dispersed in PP matrix and served multiple roles by tuning melt viscoelasticity for improving microcellular foaming behavior, enhancing crystallization of PP matrix and contributing to improving the uniformity of PDPP’s dispersion in INF composite. Compared to pure PP foam, PP/PET(F)/PDPP foam exhibited refined cellular structures, thus the cell size of PP/PET(F)/PDPP foam was decreased from 69 to 23 μm, and the cell density increased from 5.4 × 10^6^ to 1.8 × 10^8^ cells/cm^3^. Furthermore, PP/PET(F)/PDPP foam showed remarkable mechanical properties, including a 975% increase in compressive stress, which was attributed to the physical entangled PET nanofibrils and refined cellular structure. Moreover, the presence of PET nanofibrils also improved the intrinsic flame-retardant nature of PDPP. The synergistical effect of the PET nanofibrillar network and low loading of PDPP additives inhibited the combustion process. These gathered advantages of PP/PET(F)/PDPP foam make it promising for lightweight, strong, and fire-retardant polymeric foams.

## 1. Introduction

With the development of plastic materials, lightweight, high strength, functional, and related advanced molding technology and instruments have become development trends in the field of plastic products processing technology [1,2]. Polypropylene (PP) is one of the most widely used thermoplastics in the plastic market due to its low cost and density, excellent processability, good chemical resistance and mechanical properties [3]. Taking the automotive field as an example, more than 20% of interior and exterior plastic components in automotive are usually prepared from PP and PP-based composites [4]. It is reported that the fuel consumption can be reduced by 6–8% with 10 wt% of the vehicle weight reduction [5]. Therefore, lightweight PP material becomes the primary method of automobile lightweight technology.

PP porous materials are a kind of polymer made up of a PP matrix with gas cells incorporated in them. Owing to their light weight, thermal and acoustic insulation, excellent thermal resistance, and high specific mechanical strength [6], PP-based foams are commercially and technologically suitable for application in many industries including automotive and aerospace, cushioning and packaging, and construction and building. At present, various technologies, including particle leaching [7], thermal phase separation [8], ultrasonic [9] and 3D printing [10] have been developed to fabricate PP porous materials. Microcellular gas foaming with supercritical carbon dioxide (scCO_2_) as a physical blowing agent is an advanced environmentally friendly and solvent-free way of manufacturing porous materials [11]. ScCO_2_ foaming becomes a promising candidate technology to fabricate PP or PP-based composites foams with enhanced mechanical and functional performances. However, there are several challenges to be solved. First, PP has low melt strength due to the fact that the linear molecular chains are easy to disentangle [12]. Thus, cell collapse and coalescence usually happen during cell growth in the scCO_2_ process [13], resulting in poor mechanical performance. Second, in the scCO_2_ batch foaming process, complex crystallization behavior of PP matrix makes it more difficult to control the cell nucleation and cell growth stage, leading to the inability to obtain a homogeneous and stable cell structure [14]. Third, for the composite foams, it is well known that the cell structure is associated with the distribution of dispersed phase or functional fillers. The mechanical properties of foamed parts are generally inferior to solid parts [15] due to the non-uniformly distributed cells acting as stress concentration points.

Previous studies have been conducted in the area of improving the foamability of PP. Strategies such as polymerization, crosslinking and branching [16], melt blending (e.g., polytetrafluoroethylene (PTFE) [17], Nylon [18]), and compounding with additives (e.g., fiber [19], talc [20], CNF [21]). The general deficiencies of these existing methods lie in the contradiction between process complexity and modification effect, as well as the dispersion of nanoparticles in polymer matrix. In situ nanofibrillation (INF) technology is recognized as a cost-effective and high-efficiency process for the fabrication of fiber reinforced composites [22]. Compared to the glass fiber or carbon fiber reinforced composites, the addition of inorganic fibers cannot induce a sufficient strain hardening effect, which is the key point to improve melt strength. In the INF process, under the combined function of hot drawing and shear flow fields, the phase morphology of dispersed phase domains changes from spherical to fibrous shape. This in situ deformation of dispersed domains can form micro/nano fibrils, and the uniform distribution of large number of fibrils is efficient for the formation of physical fiber reinforced network, which can improve the melt viscoelastic, crystallization, mechanical performances and foamability of the INF composite [23,24]. It is noteworthy that the incompatibility between the matrix and the dispersed phase reduces the interfacial adhesion. However, the weak interfacial interaction leads to the stripping of micro/nanoscale fibrils from the matrix at the interface due to the stress concentration effect, thus reducing the mechanical properties [25]. Therefore, the incorporation of functional third phase additives can not only endow the composite foams with functional properties, but also help to balance the conflicting requirements of in situ fibrillation and interfacial adhesion on compatibility demands.

Due to the organic characteristics of PP, the flammability of PP composite foam has attracted more and more attention to fire risk. The incorporation of halogen-based flame-retardant compounds (FR) in polymer matrix is one of the most effective and widely used strategies to enhance the fire resistance of PP [26]. Due to the excellent flame-retardant effect, along with low cost of characteristic, even when low loadings (<5 wt%) of halogen-based FR are introduced into polymer matrix, the impact properties loss can be minimized. Nevertheless, the FR normally cause serious environmental problems owing to the release of toxic materials [27]. So, much attention in industry and academia has focused on developing halogen-free FR. However, much higher loadings (>20 wt%) of halogen-free FR are usually needed to achieve efficient flame-retardant effect. This not only increases the costs and impairs the mechanical properties, but also decreases the microcellular foamability of polymers by reducing the melt strength. It can be explained from the depraved cell coalescence and coarsening mechanism. In this situation, more cell rupturing occurred, and cell stabilization is reduced, leading to non-uniform foams with a lower cell density and bigger cell size. Therefore, novel types of FR and related synergistic flame-retardant mechanisms are needed to be developed. Polydiphenoxyphosphazene (PDPP), one of the linear polyphosphonitriles, is an inorganic-organic hybrid macromolecule with high elasticity and excellent flame retardancy [28,29]. Its main molecular chain is alternately connected by single and double bonds in phosphorus and nitrogen atoms, and the lateral chain is replaced by double phenoxy groups [30,31]. The intumescent char layer produced by the synergistic effect of phosphorus and nitrogen during combustion, associate with the dehydration hairy effect are considered to be the dominant flame-retardant mechanisms of PDPP [32]. To the best of the authors’ knowledge, only few studies have reported the incorporation of PDPP additives which can improve the flame-retardant of polymer composite as FR [33,34], while no investigations were found regarding the effect of PDPP on the fabrication of polymer composite foams.

In this work, an effective INF technology associated with the eco-friendly microcellular foaming process was proposed for fabricating PP composite foams with the desired cellular structure and enhanced compressive properties and fire retardancy. First, PP/PET/PDPP composites with PET nanofibrils were prepared by combining twin-screw compounding with the melt spinning process. Then, the effects of the presence of PET nanofibrils on the PDPP’s distribution, foaming behavior of PP/PDPP compounds, and flame retardancy were investigated. Furthermore, the effect of PET nanofibrils on the compressive stress-strain behavior of INF foams was also studied. To characterize the prepared composite foams in terms of flammability, the combustion behavior of different samples was analyzed, it also shows self-extinguishing behavior when the amount of flame retardant is lower.

## 2. Experimental

### 2.1. Materials

An isotactic PP (homopolymer, Moplen Z30S, Maoming Petrochemical Co., Ltd., Maoming, China) was provided as a matrix polymer. It exhibited good flow characteristics, with a density of 0.9 g/cm^3^ and a melt flow rate (MFR) of 25 g/10 min (230 °C/ 2.16 kg). The dispersed polymer phase used in this research was PET (FC510, Yizheng Chemical Co., Ltd., Yizheng, China). DSC determined the melting points of PP and PET to be 167 °C and 253 °C, respectively. A commercially available PDPP in the form of white powder was used as the flame-retardant filler, supplied by Shoucheng Chemical Co., Lit. CO_2_ with a purity of 99.9% provided by Xieli Special Gas Inc., was used as the physical blowing agent.

### 2.2. INF Composite and Foams Preparation

**Figure 1:**
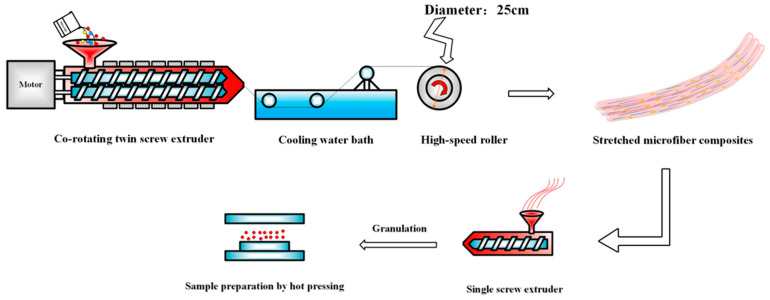
Schematic illustration of the INF composite fabrication.

An INF process combining twin-screw compounding with high-speed hot drawing was illustrated in Figure 1. Firstly, pure PP, PET pellets and PDPP powder were dried at 80 °C for 8 h to remove moisture. Melt compounding was then accomplished with a co-rotating twin-screw extruder (screw Φ22 mm, L/D = 40, Ruiya Co., Ltd., Nanjing, China). During this process, the temperature of the extruder from the feed section to the die is set at 160 °C, 190 °C, 235 °C, 265 °C, 265 °C, 265 °C and 255 °C, the screw speed is set at 75 rpm, and the feed rate is maintained at about 6.5 kg/h. The formation of pure PP and other composites were listed in Table 1.

The extrusion of the die is connected to a custom-made high-speed drawing device consisting of a variable speed motor and a Φ25 mm high-speed roller. By controlling the roller speed, the fiber with diameter of 160 μm and draw ratio of ~12.5:1 (the ratio of die to fiber diameter) was obtained. Finally, all prepared INF splines were fed into a single screw extruder for the disorientation of PET nanofibrils and subsequent granulation. The temperature of the barrel of the single screw extruder was maintained at 190 °C to keep the nanofibrils’ morphology, and the screw speed was set at 15 rmp. In order to maintain a same thermal history, pure PP, PP/PET(S), and PP/PET(S)/PDPP samples followed the same process mentioned above. Many trials in prior to formal experiments have been optimized to acquire the above processing settings.

All prepared INF composites were foamed based on a customized batch microcellular foaming unit with scCO_2_ as physical blowing agent. Rectangular samples (length 20 mm × width 5 mm × thickness 4 mm) were first placed inside the chamber and the CO_2_ was pressurized to 3000 Psi using a high-pressure syringe pump (ISCO-260D, Global Technology Co., Ltd., Lincoln, NE, USA). The saturation temperature of 145 °C was set as foaming temperature. Subsequently, the foaming chamber was pressurized for 1.0 h to ensure the CO_2_ was entirely diffused and dissolved in samples. After achieving polymer/scCO_2_ saturation, cell nucleation and cell growth were occurred by inducing thermodynamic instability by rapidly releasing the gas from the chamber within 2 s. Stabilization of the cell structures was then formed by putting the chamber into an ice-water bath.

### 2.3. Characterization

#### 2.3.1. INF Composites and Foams Morphology Characterization

The morphology of the composites before and after INF process was observed by scanning electron microscopy (SEM, Tuscan Mira LMS, Brno-Kohoutovice, Czechia). Prior to the examination, the unstretched sample was first fractured in cryogenic liquid nitrogen and sputtered with platinum on the surface. In order to study the morphological evolution of the PET domain, INF samples were placed in xylene vapor at 140 °C for 2.5 h, and then ultrasonic cleaned twice in deionized water to remove the PP matrix. All prepared samples were then dried and sputtered with platinum.

The cell morphologies of foamed samples were characterized by SEM equipped with an energy-dispersive X-ray spectroscopy (EDS) detector. All foamed samples were immersed in liquid nitrogen for at least 10 min and then coated with platinum by sputtering. SEM micrographs were captured from randomly selected regions. Quantitative characterizations of the cellular structures were carried out by measuring the expansion ratio (*φ*), cell size, cell density (*N_f_*), and cell size distribution.

According to ASTM D792, the *φ* values of all foam samples are determined by the drainage method. *φ* is defined associated with solid density (*ρ_s_*) and foam’s density (*ρ_f_*):

(1) $$\varphi =\frac{\rho_{s}}{\rho_{f}}$$

Cell density *N_f_* is defined as the number of cells per unit volume of a foamed sample. *N_f_* was obtained by analyzing the SEM micrographs of the foamed samples using the Image-Pro Plus software, and was calculated using the Equation (2):

(2) $$N_{f}={\left(\frac{NM^{2}}{A}\right)}^{3/2}\times \varphi$$

where *N* is the number of cells counted in the area *A* (cm^2^) for a selected SEM micrograph. *M* is the magnification factor of SEM micrograph. At least 200 cells were contained in area *A* to obtain statistically reliable data.

#### 2.3.2. Rheology

A dynamic strain sweep was first performed using a rotational rheometer (DHR-2, TA) to identify the linear viscoelastic region of all samples. Subsequently, a dynamic frequency sweep was conducted with a frequency (ω) ranging from 100 to 0.01 rad/s at 190 °C to measure the storage modulus (G′), loss factor (tan δ), and complex viscosity (η*). To prevent oxidative degradation of the sample, all tests were conducted under the protection of nitrogen atmosphere.

#### 2.3.3. Non-Isothermal Crystallization

Differential scanning calorimetry (DSC, Q2000, TA, New Castle, DE, USA) was utilized to characterize the non-isothermal crystallization behaviors of pure PP and PP-based composites under a nitrogen atmosphere at 50 mL/min. All samples were equilibrated from 40 °C to 200 °C with a heating rate of 10 °C/min and kept for 5 min to remove the thermal and stress history, then cooled to 40 °C at 10 °C /min to measure the crystallization behavior, subsequently heated at 10 °C/min to 200 °C to obtain the melt behavior. The relative crystallinity $\chi_{c}$ of samples was calculated by Equation (3):

(3) $$\chi_{c}=\frac{\Delta H_{m}}{\Delta H_{m}^{0}\cdot f}$$

where $\Delta H_{m}$ represents the fusion enthalpy crystallization of samples (J/g). $\Delta H_{m}^{0}$ is the crystallization enthalpy of 100% crystallized PP (209 J/g). *f* is the weight fraction of PP in each composite.

#### 2.3.4. Uniaxial Compression

Uniaxial compression tests of INF composite foams were performed on cubic specimens with a size of 5 mm × 5 mm × 5 mm using a UTM 2203 testing machine equipped with a 100 N load based on ASTM D1621-2010 (Selangor, Malaysia). The maximum compression strain was set to 60% and the compression rate was set at 1 mm/min. To minimize the test error, at least three specimens were tested, and the average value was reported.

#### 2.3.5. Flame Retardancy Characterization

A horizontal and vertical combustion instrument (CZF-3, Beijing Zhonghang Times Instrument Equipment Co., Ltd., Beijing, China) was employed to compare the flame retardancy of the neat PP, and PP based composites. The horizontal UL-94 tests were conducted according to ASTM D3801, and the dimensions of the sample size were 125 mm× 12 mm × 4 mm. Then, the experiment was carried out in a windless environment with high-definition cameras, alcohol lamps, a fire starter, tweezers, and cotton wool. In the whole experiment, high-definition digital cameras were used to shoot the combustion process of different foamed samples after ignition, recording the moment when the samples were ignited, whether the dripping melt would ignite the absorbent cotton and the final combustion behavior of the samples.

## 3. Results and Discussion

### 3.1. INF Composite Morphology

**Figure 2:**
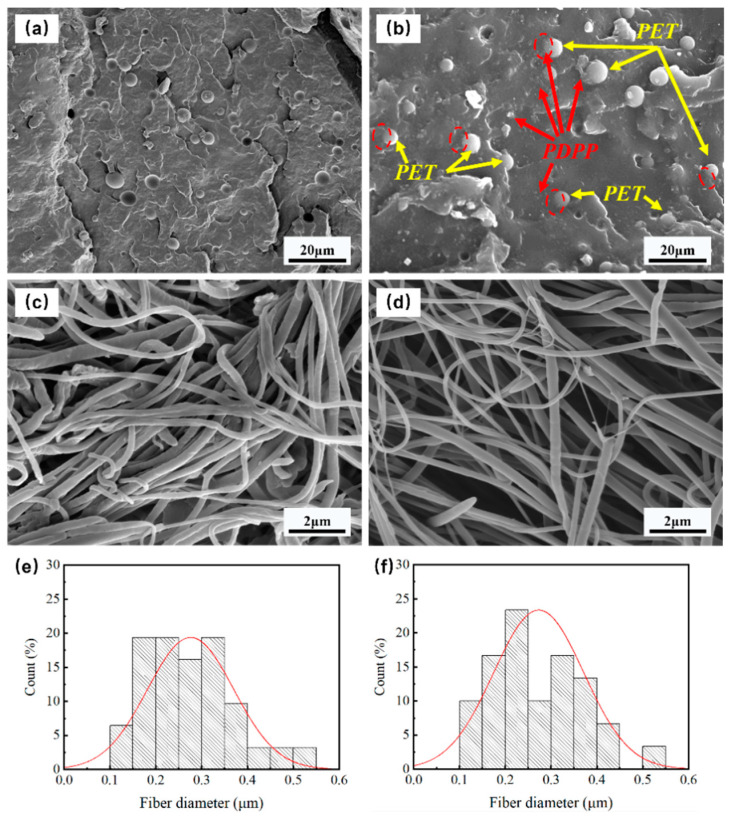
Micrographs of the melt-blended composites PP/PET(S) (**a**) and PP/PET(S)/PDPP (**b**). SEM images of INF composites PP/PTE (F) (**c**), PP/PET(F)/PDPP (**d**) after etching PP matrix, and corresponding size distribution of PET nanofibrils (**e**,**f**).

The phase morphologies of all PP-based composites are depicted in Figure 2. It can be found from Figure 2a, b that both micrographs exhibit typical “sea-island” phase morphologies, and the PET spherical domain is uniformly dispersed in the PP matrix. Compared with PP/PET(S) blends, it is clear that the phase interface between the PP matrix and the spherical PET domain in the PP/PET(S)/PDPP composite becomes rougher, and fewer PET spherical domains are stripped. Furthermore, after adding the same amount of PDPP additives (Figure 2b), no agglomeration phenomenon is detected, and many small white particles distributed in the PP-PET interface and PP matrix (Tables S1–S3). The selective dispersion of PDPP particles at the PP-PET interface appears to play a role in interfacial compatibilization [34]. Figure 2c,d illustrates the morphologies of INF composites after the PP matrix is etched with xylene. It is evident that the PET domains have been fibrillated into an entangled fibril network with nanoscale diameter. Figure 2d obviously depicts almost the same size of PET nanofibrils, but a bigger length-diameter ratio in comparison to Figure 2c. The size distribution of PET nanofibers is shown in Figure 2e,f to compare the influence of PDPP particles on the diameter of PET fibrils. Compared with PP/PET(F) composite, the average diameter of PET fibrils remains at 270 nm, but the proportion of the fibrils distributed in 100~250 μm is increased. These results indicate that the incorporation of PDPP particles has no negative effect on the formation of PET nanofibrils and can generally effectively improve the microcellular foaming properties [35].

### 3.2. Rheological Behavior

**Figure 3:**
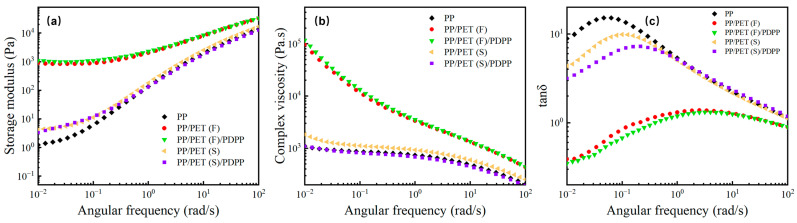
Rheological properties of the neat PP, melt-blended, and INF composites: (**a**) storage modulus (G′), (**b**) complex viscosity (η*), and (**c**) loss tangent (tan δ).

The rheological property of polymer composite has a significant effect on foaming behavior [36]. Figure 3 illustrates the relationship between storage modulus (G′), complex viscosity (η*) and loss tangent (tan δ) with frequency (ω). As can be seen from Figure 3a that the INF composites displayed an increment of G′ at the whole frequency and a plateau occurred at low frequency. Remarkably, the slope of G′ curve for INF composites were about 1 at low frequencies region, which is smaller than that of pure PP and melt-blended composites. The shift of G′ can be attributed to the inhibition effect owing to the presence of PET nanofibrillar physical network and increased entanglements between PET nanofibrils and PP molecular chains [37,38]. Furthermore, for comparison, the viscoelasticity of PP/PDPP composite was also carried out to determine the effect of PDPP particles on viscoelasticity of PP matrix (Figure S1). It was found that the viscoelasticity of PP matrix decreased after adding PDPP individually due to the plasticization effect [33], which can impair the PP’s microcellular foamability. However, G′ of PP/PET(F)/PDPP is slightly higher than that of PP/PET(F) due to the higher length-diameter ratio of PET nanofibrils, which further increases the PP molecular chain’s entanglement. This result is in good agreement with the results of phase morphology discussed before. Figure 3b illustrates that both INF composites presented similar typical non-Newtonian fluid behaviors and more obvious shear shinning effect than that of other samples. η* of INF composites is significantly higher than that of pure PP and melt-blended composites, particularly in the low frequency region. It can be explained that the PET fibril network can restrict the motion of PP molecular chains as a role of a skeleton within PP matrix [36]. It is noteworthy that the η* of PP/PET(S) composite decreased greatly after the introduction of PDPP particles, while the η* is almost kept constant for both INF composites. This demonstrates that the formation of PET fibril network can effectively compensate the negative effect of PDPP on the viscosity of PP matrix.

Figure 3c depicts the curve of tan δ as a function of frequency. Both pure PP and melt-blended samples exhibit a typical viscoelastic liquid with one peak in the low frequency region, followed by a downward trend in the frequency region greater than 0.1 rad /s. Meanwhile, the tan δ value is bigger than 1. However, both INF composites show a slow increase in tan δ with increasing ω, until the high frequency region (>10 rad/s) tan δ trend to decrease. The transition of a liquid-like behavior to a solid-like behavior can be attributed to the formation of PET fibril network, which can sharply reduce viscous dissipation tan δ [39].

### 3.3. Crystallization Behavior

**Figure 4:**
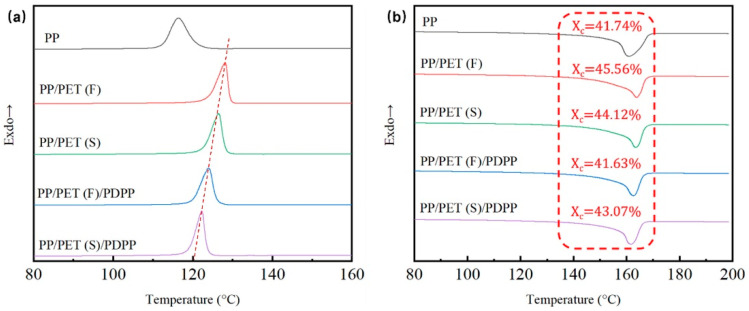
Cooling (**a**) and melting (**b**) curves of diverse PP-based composite samples.

Owing to the state of the crystals in semi-crystalline polymer significantly affects the microcellular foaming process, it is necessary to test the crystallization properties of composites. The melting and cooling DSC curves of various pp matrix composites are shown in Figure 4. It can be observed from Figure 4a that the initial crystallization peak temperatures (Tc) of all PP-based composites were much higher than that of pure PP due to the nucleation effect of heterogeneous crystallization. The free energy barrier was lowered, and the crystal nucleus are easily formed [40]. Furthermore, each INF composite have higher Tc than that of the corresponding melt-blended sample. It reveals that the PET nanofibrils can promote the crystallization rate of PP matrix sharply due to its large specific surface areas, which can act as heterogeneous crystal-nucleating agents [41]. After the introduction of PDPP, Tc shifted to low temperature, and even as low as the pure PP for PP/PDPP blend (Figure S2). This can be explained from the PDPP’s plasticization effect. PDPP particles are inserted into PP molecular chains which weakens the interaction force among molecular chains, resulting in promoting the mobility of PP molecular chains and subsequently decreasing the Tc [33].

To further investigate the effect of PET fibrils and PDPP particles on PP’s crystallinity, non-isothermal melting thermograph of samples were shown in Figure 4b. Melting peak temperatures (Tm) were calculated and labelled. The maximum crystallinity 45.6% and minimum 41.6% were obtained for PP/PET(F) and PP/PET(F)/PDPP composites respectively. According to the CO_2_ adsorption tests for INF composites (Figure S3), it can be found that polymer composite with higher crystallinity exhibited lower CO_2_ concentration because it difficult for scCO_2_ dissolving into the amorphous region, thus resulting in lower expansion rate [42,43]. Additionally, the crystal melting behavior primarily occurs at temperatures ranging from 155 to 168 °C, and the crystallization triggering at 115 to 128 °C for all samples. It is well known that scCO_2_ normally can decrease Tm [44], so the foaming temperature for PP-based composites can be considered in the range of 130~150 °C.

### 3.4. Cellular Morphology

To explore the effect of PET fibrils and PDPP particles on the microcellular foaming behavior of PP matrix, the cellular morphologies of the pure PP and PP-based composite foams were observed. Since the incorporation of PDPP particles can lead to very low melt viscoelasticity and Tc value (Figures S1 and S2), the PP/PDPP and PP/PET(S)/PDPP foams show very low expansion ratio (<2.0) and seriously deteriorated cell structures with cell collapse (Figure S4).

**Figure 5:**
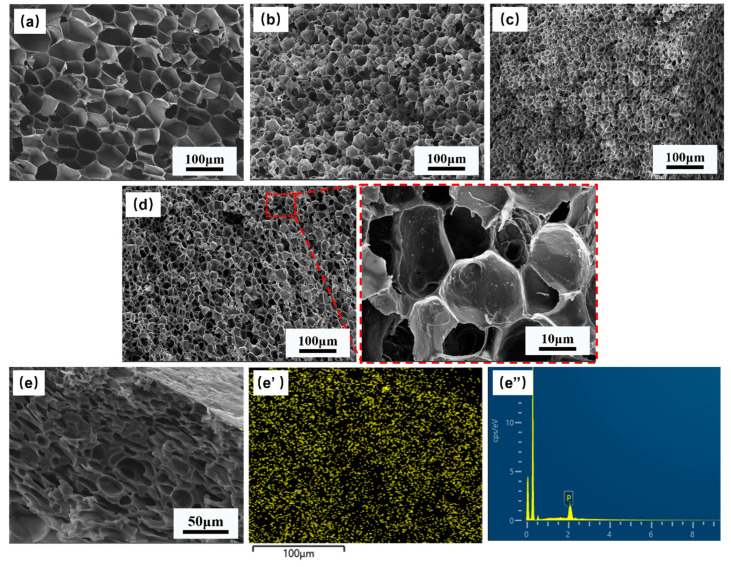
Cell morphology of different foams and dispersion mechanism of PDPP: (**a**) neat PP; (**b**) PP/PET(S); (**c**) PP/PET(F); (**d**,**e**) PP/PET(F)/PDPP; (**e**’) EDS mapping of P element; (**e**”) EDS spectrum.

**Figure 6:**
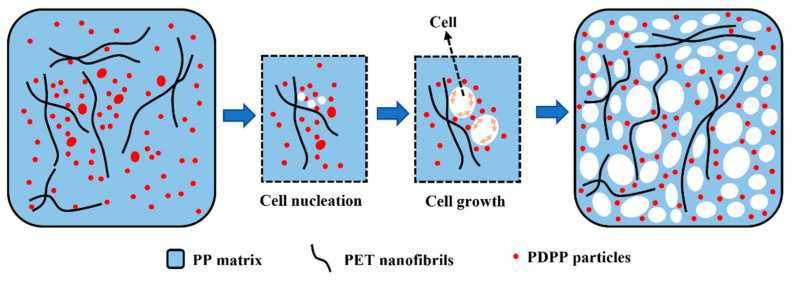
Schematic diagram of the dispersion mechanism of PDPP during the growth of cell.

Figure 5a,d shows the cellular morphologies of the pure PP, PP/PET(S), PP/PET(F) and PP/PET(F)/PDPP foams prepared at 145 °C under 3000 Psi of pressure for 1 h to maintain the PET’s fibrillar shape and solid state. Pure PP exhibits huge, regular-like polygonal shapes with closed cell structures. The introduction of spherical PET domains endows a reduction in cell size, an increase in cell density, and an improvement in cell homogeneity due to the heterogeneous cell nucleation effect [35]. After PET domains transformed from spherical droplets to fibrils, the cell size became much smaller and cell density increased dramatically. This improvement can be ascribed to the larger cell-nucleation driving force provided by the PET fibrils with a large specific surface area to overcome the higher cell-nucleation elastic energy barrier [41]. Moreover, as discussed before, the enhancement in viscoelasticity and increase of Tc can improve the cell wall’s resistance to deformation during cell growth stage. The mechanism diagram of microcellular foaming process for INF composite is also depicted in Figure 6. Moreover, it is evident from Figure 5d that the cellular morphology of PP/PET(F)/PDPP foam exhibits no big difference from that of PP/PET(F) foam. Only slightly increased cell size and decreased cell density were obtained. This is mainly attributed to the PDPP’s plasticization, which results in weaker melt strength and lower efficiency in cell growth. It is interesting to note that some PET fibrils were observed on the cell wall, and PDPP particles were also detected to be uniformly dispersed on the cell wall without obvious agglomeration. This can bring about an efficient improvement in flame-retardant performance. Moreover, the energy dispersive spectrum (EDS) was utilized to examine the dispersion of P element within the INF foams to better comprehend the selective dispersion of PDPP particles. Element mapping results and EDS spectrum were indicated in Figure 5e,e’, respectively to examine the cross-section of INF composite foams. It was confirmed that a large amount of PDPP particles uniformly distributed both in the cell wall and nonporous bulk layer. These reasons are attributed to the synergetic effect of both PDPP plasticization and bidirectional stretching during cell growth. Previous studies have indicated that scCO_2_ can reduce the viscosity of polymer melt, increase the mass transfer rate and solubility in polymer due to its plasticization effect [45,46]. As illustrated in Figure 6, with the diffusion of CO_2_ during cell-growing, the biaxial stretching effect of cell expansion on polymer matrix contributes to the dispersion of PDPP particles moving to the surface of the foamed material [47]. Therefore, the gathered results not only strongly support the promoting effect of scCO_2_ on particle dispersion, but also demonstrate that the excellent cellular structures can be obtained by the introduction of PET fibrils.

**Figure 7:**
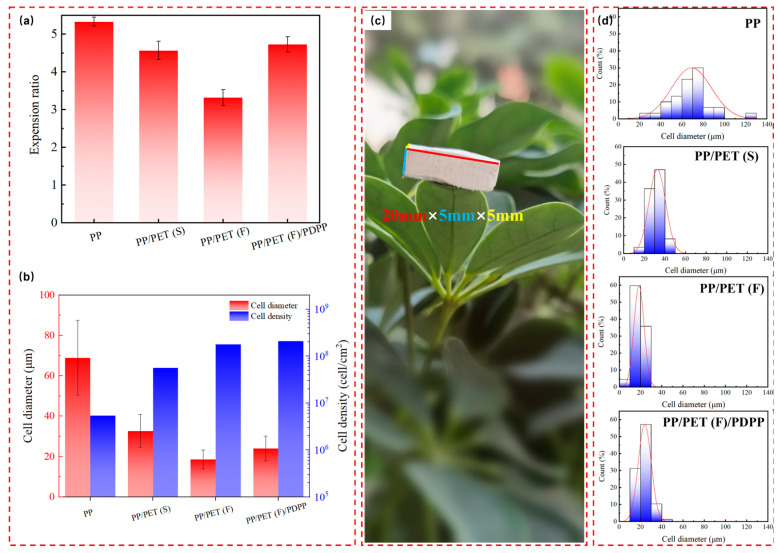
(**a**) Expansion ratio; (**b**) average cell diameter and cell density; (**c**) photograph of PP/PET(F)/PDPP foam placed on plant leave, and (**d**) cell size distribution of neat PP, PP/PET(F) and PP/PET(F)/PDPP foams.

As shown in Figure 7a–c, expansion ratio, cell diameter, cell density and cell distribution were determined to quantitatively characterize the cellular structures. High expansion ratio can provide lightweight polymer foams. Figure 7a shows the comparison of the expansion ratios of pure PP and PP-based composite foams. The minimum expansion ratio 3.3 is obtained from PP/PET(F) foam, reducing by 36% compared to pure PP foam. One reason may due to that the presence of PET fibrils increases the melt strength strongly and constrains the cell expansion [48]. Another reason is that the high crystallinity of PP/PET(F) composite makes it difficult for CO_2_ to dissolve in the amorphous region of the molecular chain, thus reducing the cell nucleation efficiency (Figure S3). After the introduction of PDPP particles in INF composite, the crystallinity decreases and more CO_2_ absorption is obtained [49], resulting in an increase in the expansion ratio from 3.3 to 4.7. Figure 7b displays the average cell size and cell density of foams. With the morphologies of PET domains changed from spherical to fibril shape, the cell size decreased obviously due to the heterogeneous nucleation effects and the improvement of melt strength caused by PET nanofibrillar network. The cell size of PP/PET(F) foams drastically decreases from 69.0 μm to 18.6 μm, and the cell density was increased by two orders of magnitude from 5.4 × 10^6^ cells/cm^3^ to 1.8 × 10^8^ cells/cm^3^ in comparison to pure PP foams. In contrast, adding 5 wt% PDPP increases the cell size of PP/PET(F)/PDPP foams by the amplitudes of 67%, which is caused by the weak local melt strength due to the low crystallinity. However, the cell density almost keep constant because of the same cell nucleation ability. Additionally, the cell size distribution of foams is also depicted in Figure 7d. It can be observed that the narrowest cell size distribution range was obtained for INF foams, and the range of cell size distribution decreased from 20~140 μm to 8~40 μm when compared to PP foams. In general, PET nanofibrils can compensate for the detrimental effect of PDPP particles on microcellular foamability of PP matrix. By using fabrication technology suggested in this work, lightweight INF foams with a dense and uniform cellular structure was attainable (Figure 7c).

### 3.5. Compressive Property

**Figure 8:**
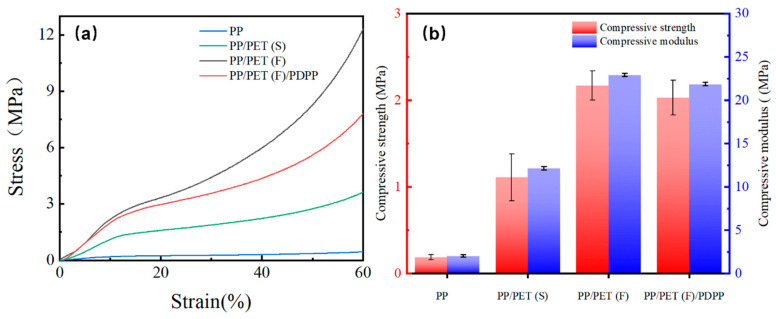
(**a**) Compressive stress–strain curves, and (**b**) compressive strength and compressive modulus, for pure PP, PP/PET(S), PP/PET(F) and PP/PET(F)/PDPP foams.

It is known that the cell structures significantly affect the mechanical properties of polymer foams [50]. Considering the application conditions, compression tests of prepared foams were performed. Figure 8a exhibits the compressive stress as a function of compressive strain, and Figure 8b compares the obtained compressive strength and modulus for all foamed samples. It can be clearly seen that all curves consist of three typical stages of deformation: (i) linear elastic deformation; (ii) plateau of plastic deformation; and (iii) densification behavior which is attributed to the collapse of the cell walls, showing a sharp and irreversible stress increase [51]. It is noteworthy that the range of plastic deformation plateau for PP/PET(F)/PDPP foams is larger than that of PP/PET(F) foam, which can be understood as a higher expansion rate can extend the yielding process during the compression stage under the condition of similar cell size and density. By comparing the values of compressive strength and modulus in Figure 8b, it can be found that incorporating 5 wt% spherical PET domains to PP led to dramatic increases in compressive strength of PP from 0.2 MPa to 1.1 MPa, and the modulus also improved from 2.4 MPa to 12.4 Mpa. However, the presence of PET nanofibrils in the PP matrix further enhances the mechanical stress and modulus. The compressive stress and modulus increased by 975% and 858%, respectively. It can be explained by two reasons: (i) the presence of physical entangled PET nanofibrils with high aspect ratio can provide a high specific area, resulting in promoting the stress transfer between PP matrix and reinforcement phases [52]. (ii) As analyzed in the previous section, PP/PET(F) exhibited the smallest cell size comparing other foams. It has been well accepted that the smaller cell size can substantially increase the stiffness of foams [48,53]. Although introducing PDPP particles into PP/PET(F) led to a slight reduction in compressive stress and modulus, it is effective to obtain such a foam with improved compressive stress, stiffness and long range of plastic deformation regions with the compensation of PET nanofibrils.

### 3.6. Flame Retardancy Property

**Figure 9:**
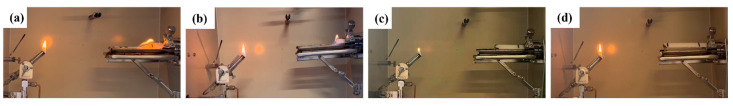
The photograph of horizontal UL-94 combustion testing for solid samples (**a**) neat PP, (**b**) PP/PET(F), (**c**) PP/PET(S)/PDPP and (**d**) PP/PET(F)/PDPP.

Figure 9 depicts the neat PP, PP/PET(F), PP/PET(S)/PDPP, and PP/PET(F)/PDPP composites during horizontal UL-94 combustion measurements. Table 2 lists the measured combustion rates and UL-94 levels. It is not difficult to find that the horizontal combustion levels of PP and PP/PET(F) are FH-3-20 and FH-3-21. By contrast, PP/PET(S)/PDPP and PP/PET(F)/PDPP reach the flame retardancy level of FH-2-23 mm and FH-1, respectively. It can be explained that the PDPP particles during combustion will produce a variety of flame retardant gas isolation air and heat, HPO, PO_2_· and other free radicals, which can inhibit the combustion of polymer materials. PP/PET(F)/PDPP composite reaches the highest UL 94 level combustion rating, indicating that the presence of PET nanofibrils can improve the dispersion of PDPP. Similar results were also achieved in another article [54].

**Figure 10:**
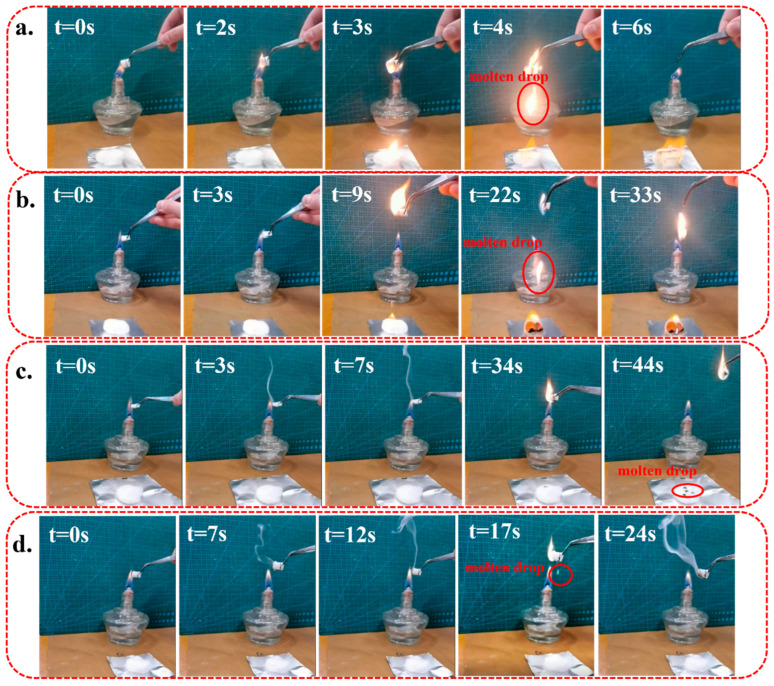
The snapshots of combustion behavior of (**a**) PP, (**b**) PP/PET(F), (**c**) PP/PET(S)/PDPP, and (**d**) PP/PET(F)/PDPP foams.

The combustion behaviors of different foams were compared and investigated. Figure 10 shows the photos of foam combustion (Videos S1–S4). It can be seen that pure PP and PP/PET(F) foams are easy to ignite, burning rapidly and violently. In the initial combustion stage, continuous dripping of molten droplets occurs in the initial combustion stage, and many of them drop on the surface of the cotton, leaving no residue. The molten droplets easily ignite the cotton and cause the spread of fire. In contrast, PP-based foams with PDPP particles are ignited after several attempts. During the combusting process, foamed samples with PDPP have small combustion flame and they can immediately self-extinguish after being removed from the fire source. Some white fog production is detected which is maybe the water vapor produced by dehydration in the decomposition of PDPP [32]. Unlike the dripping of molten drips, which happened in the initial stage of combustion shown in Figure 10a,b, some water-like flameless droplets occur in the second half of combustion as depicted in Figure 10c,d. Instead of igniting the cotton, both PP-based foams with PDPP additives can carry away the heat generated by the combustion until the foam self-extinguishes. On further comparison between combustion features of PP/PET(S)/PDPP foam and PP/PET(F)/PDPP foam, it is observed that PP/PET(F)/PDPP foam characterized as more ignition times and shorter self-extinguishing time. This demonstrated that compared to PP foams without PDPP or PET nanofibrils, PP/PET(F)/PDPP foams have excellent flame retardant and molten drops combustion suppression performance, so they can reduce fire risk and fire hazards.

**Figure 11:**
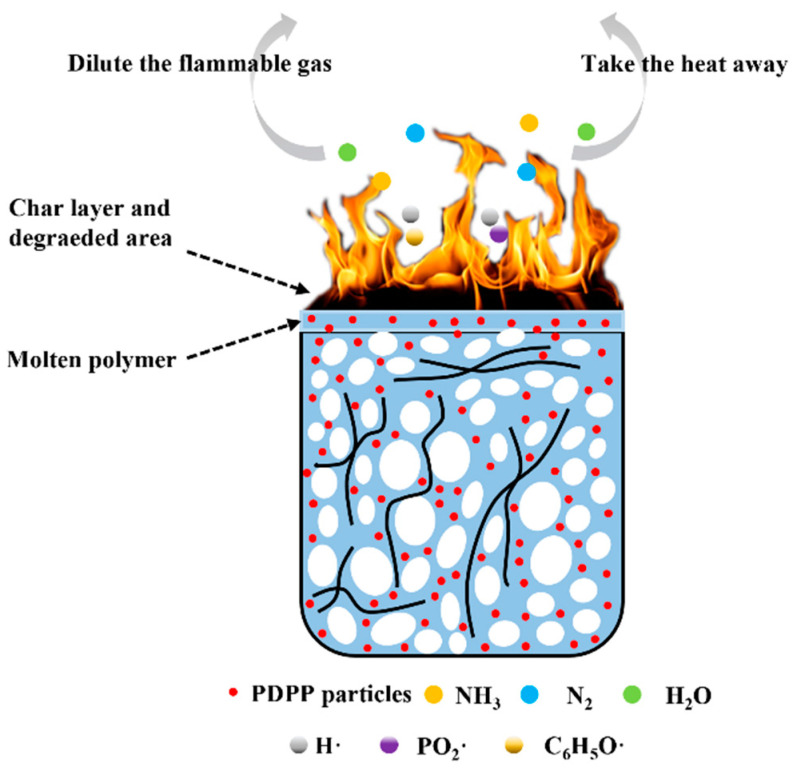
Flame retardant mechanism of PP/PET(F)/PDPP foam.

The flame-retardant mechanism of PP/PET(F)/PDPP foam is also shown in Figure 11. The synergistic flame-retardant effect of PDPP particles and PET nanofibrils and the generation of a compact carbon-containing char layer on the surface could prevent the transfer of combustible volatiles from the matrix to the flame, thereby protecting the foams from burning [55,56]. Firstly, it is well known that the scCO_2_ foaming process is efficient for the uniform dispersion of PDPP particles due to the gas-like diffusivity of scCO_2_ [57], which can obtain favorable flame-retardant performance. During the combustion process, PDPP particles on the cell surface are decomposed into phosphoric acid, metaphosphate and polyphosphate, which have strong dehydration characteristics [32]. The pyrolysis of PDPP is recognized as an endothermic process, and some non-flammable gases, such as ammonia, nitrogen, and water vapor can dilute the concentration of combustible gases and take away most of the heat [32,33]. That is why the PDPP particles can play an important role in the flame retardation of gas phase during combustion. Additionally, PDPP particles can release HPO, PO_2_·, C_6_H_4_O, which can combine with H· and HO· free radicals known as the main reactants in the thermal oxidation chain reaction, and finally inhibiting the combustion reactions of polymers in the gas phase [58]. Finally, the melt strength of PP matrix was greatly improved by PET nanofibrils, which resulted in a significant reduction of flameless droplets in water samples during combustion [59]. These gathered results indicate that INF composite foams with PDPP particles have much better flame retardancy than pure PP and other PP-based composite foams for thermal insulation.

## 4. Conclusions

In summary, the lightweight and strong PP-based composite foams with favorable flame retardancy were successfully achieved by combining in situ nanofibrillation (INF) and scCO_2_ microcellular foaming processes. The nanofibrillar PP/PET/PDPP composite was firstly manufactured by conventional twin-screw compounding with melt spinning. PET nanofibrils with an average diameter of 270 nm were produced, and incorporation of PDPP particles had no negative effect on the size of PET nanofibrils and improved the uniformity of fibrils’ distribution. Owing to the incorporation of PET nanofibrils and PDPP particles, the melt strength and crystallization of the PP matrix were significantly enhanced, which in turn improved the foamability during scCO_2_ microcellular foaming. Compared to pure PP foam, the cell structures were refined, thus the cell size of PP/PET(F)/PDPP foam was decreased by 67%, from to 69 to 23 μm, and the cell density increased by two orders of magnitude, from 10^6^ to 10^8^ cells/cm^3^. More importantly, the PP/PET(F)/PDPP foam exhibited significantly improved compressive property in comparison with PP foam. Its compressive stress and modulus increased by 975% and 858%, respectively. It is speculated that the presence of physical entangled PET nanofibrils with high aspect ratio can promote the stress transfer. Although PDPP can impair the matrix due to its plasticizing effect, the refined cellular structure can delay breakage by reducing the stress concentration. Furthermore, the synergistic flame-retardant effect of PDPP and PET nanofibrils was responsible for the advanced flame-retardant performance of the PP/PET(F)/PDPP foam when compared with that of pure PP foam. The presence of PET nanofibrils is efficient for the uniform dispersion of PDPP, and the dewatering function of PDPP was enhanced, resulting in the inhibition of the foam’s combustion, and the generated water-like flameless droplets cannot ignite the degreasing cotton. Therefore, the cost-effective and eco-friendly method provided in this research can provide a new option for manufacturing remarkably strong composite foams with improved flame retardancy for applications in PP-based polymeric structural components.

## Figures and Tables

**Table 1:** Formulations of pure PP and PP based composites.

Denotation	PET Content (wt.%)	PDPP Content (wt.%)	Remark
PP	0	0	Pure PP pellet
PP/PET(S)	7	0	Melt-blended PP/PET with spherical PET domains
PP/PET(S)/PDPP	7	5	Melt-blended PP/PET/PDPP with spherical PET domains
PP/PET(F)	7	0	In situ nanofibrillar PP/PET composite
PP/PET(F)/PDPP	7	5	In situ nanofibrillar PP/PET/PDPP composite

**Table 2:** The results of horizontal UL-94 tests of solid PP and PP-based composites.

Sample	Combustion Speed (mm/min)	Level
PP	19.8 ± 3.5	FH-3-20 mm/min
PP/PET(F)	21.4 ± 2.3	FH-3-21 mm/min
PP/PET(S)/PDPP	7.4 ± 2.6	FH-2-23 mm
PP/PET(F)/PDPP	-	FH-1

## Data Availability

The data presented in this study are available on request from the corresponding authors.

## Supplementary Materials

The following supporting information can be downloaded at: https://www.mdpi.com/article/10.3390/polym15061497/s1, Table S1: Contact angel value of PP, PET and PDPP at 25 °C; Table S2: Surface tension value of PP, PET and PDPP; Table S3: Interfacial tension of PP/PET, PP/PDPP, PET/PDPP. Figure S1: Effects of PDPP on viscoelastic of PP matrix; Figure S2: Effect of PDPP on crystallization behavior of PP matrix; Figure S3: Effect of PET nanofibrils and PDPP particles on CO_2_ adsorption; Figure S4: Cellular morphologies of pure PP, PP/PDPP and PP/PET(S) /PDPP foams. Video S1: Video of combustion test for pure PP foam; Video S2: Video of combustion test for PP/PET(F) composite foam; Video S3: Video of combustion test for PP/PET(S)/PDPP composite foam; Video S4: Video of combustion test for PP/PET(F)/PDPP composite foam.
