# Supplementary material for: In Situ Nanofibrillar Polypropylene-Based Composite Microcellular Foams with Enhanced Mechanical and Flame-Retardant Performances
Source: Polymers (Basel). 2023 Mar 17;15(6):1497. doi: 10.3390/polym15061497 (PMC10056583; doi:10.3390/polym15061497)
Supplement: Supplementary file 1 [file polymers-15-01497-s001.zip › polymers-2265637-supplementary.zip!/polymers-2265637-supplementary.pdf]

polymers

MDPI

SUPPORTING INFORMATION

In Situ Nanofibrillar Polypropylene-Based Composite Microcellular Foams with Enhanced Mechanical and Flame-Retardant Performances

Yufan Jiang [1,2], Jing Jiang [3,*], Lian Yang [3], Yihe Zhang [3], Xiaofeng Wang [2], Na Zhao [2], Jianhua Hou [2,*] and Qian Li [1,2]

1 School of Materials Science & Engineering, Zhengzhou University, Zhengzhou 450001, China;
2 School of Mechanics and Safety Engineering, National Center for International Research of Micro-Nano Molding Technology, Zhengzhou University, Zhengzhou 450001, China
3 School of Mechanical & Power Engineering, Zhengzhou University, Zhengzhou 450001, China
* Correspondence: jiangjing@zzu.edu.cn (J.J.); houjianhua@zzu.edu.cn (J.H.); Tel.: +86-15617960455 (J.J.)

1. The selective dispersion of PDPP in INF composites

Table S1 shows the contact angles of water and ethylene glycol on the surface of PP, PET and PDPP samples measured by the pendant drop method, and the corresponding surface tension and interfacial tension are calculated by formula (1-4) as shown in Table 1.2, 1.3 shown.

$$(1+\cos\theta_{H_2O})\gamma_{H_2O} = 4\left(\frac{\gamma_{H_2O}^{d}\gamma^{d}}{\gamma_{H_2O}^{d}+\gamma^{d}} + \frac{\gamma_{H_2O}^{p}\gamma^{p}}{\gamma_{H_2O}^{p}+\gamma^{p}}\right) \quad (1)$$

$$(1+\cos\theta_{EG})\gamma_{EG} = 4\left(\frac{\gamma_{EG}^{d}\gamma^{d}}{\gamma_{EG}^{d}+\gamma^{d}} + \frac{\gamma_{EG}^{p}\gamma^{p}}{\gamma_{EG}^{p}+\gamma^{p}}\right) \quad (2)$$

$$\gamma = \gamma^{d} + \gamma^{p} \quad (3)$$

$$\lambda_{AB} = \gamma_A + \gamma_B - 4\left(\frac{\gamma_A^{d}\gamma_B^{d}}{\gamma_A^{d}+\gamma_B^{d}} + \frac{\gamma_A^{p}\gamma_B^{p}}{\gamma_A^{p}+\gamma_B^{p}}\right) \quad (4)$$

where θ is the contact angle of water or ethylene glycol on the sample surface. γ, γ_d and γ_p are the surface tension of the sample and the dispersive and polar components of the surface tension, respectively, and λ_{AB} is the interfacial tension between the two components. It is known from the literature that the relevant values for water and diiodomethane are: $\gamma_{H_2O}^{d} = 21.8\ mN/m$, $\gamma_{H_2O}^{p} = 51\ mN/m$, $\gamma_{EG}^{d} = 29.3\ mN/m$, $\gamma_{EG}^{p} = 19\ mN/m$.

Table S1. Contact angel value of PP, PET and PDPP at 25 °C.

Sample	Water contact angle (°)	Ethylene glycol contact angle(°)
PP	95	61.5
PET	86	63.5
PDPP	85	55.5

Table S2. Surface tension value of PP, PET and PDPP.

Sample	γ^d (mN/m)	γ^p (mN/m)	γ (mN/m)
PP	24.15	6.12	30.27
PET	13.98	13.96	27.94
PDPP	35.28	3.49	38.77

Table S3. Interfacial tension of PP/PET, PP/PDPP, PET/PDPP.

Sample	Interfacial tension（*mN*/m）
PP/PET	5.77
PP/PDPP	2.80
PET/PDPP	8.61

From the measured contact Angle, the interfacial tension between different materials can be calculated by the formula of interfacial tension. Then the dispersion of PDPP in the material is predicted by the Harkin's dispersion coefficient formula.

$$\lambda_{BC} = \gamma_{AC} - \gamma_{AB} - \gamma_{BC} \quad (5)$$

$$\lambda_{CB} = \gamma_{AB} - \gamma_{AC} - \gamma_{BC} \quad (6)$$

$$\lambda_{AC} = \gamma_{BC} - \gamma_{AB} - \gamma_{AC} \quad (7)$$

A, B, C in the above formula represent PP, PET, PDPP respectively. According to the formula (5-7): $\lambda_{BC} < 0$、$\lambda_{CB} < 0$、$\lambda_{AC} > 0$, The related literature shows that if both λ_{BC} and λ_{CB} are negative, the two minor components form separate dispersed phases in the matrix. In this case, if λ_{AC} is a positive value, one component will partially encapsulate the other when a certain dispersed phase content is sufficient. S2. Effects of PDPP on viscoelastic of PP matrix

2. Effect of PDPP content on rheological behavior of INF composites

Figure S1 describes the changes of energy storage modulus and complex viscosity before and after adding PDPP into pure PP, it can be obviously observed that the energy storage modulus and complex viscosity of PP/PDPP composite decrease to different degrees after PDPP is added.

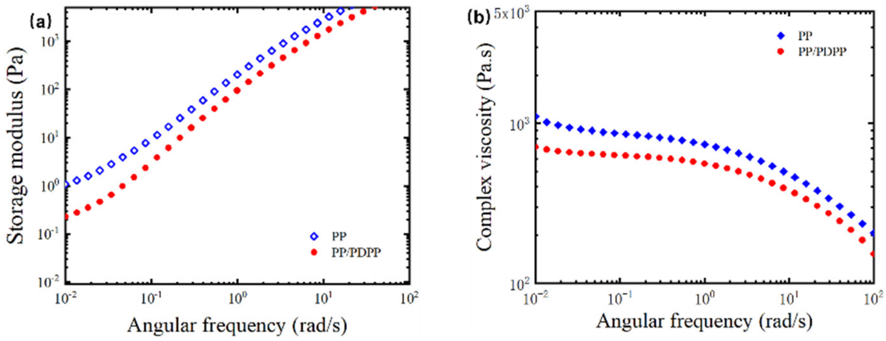

Figure S1. (a) Storage modulus (*G*′) and (b) complex viscosity (*η**) of pure PP and PP/PDPP composite.

3. Effect of PDPP on crystallization behavior of PP matrix

Figure S2 shows the heat flow curve of PP and PP/PDPP composites tested by DSC. It can be seen that the initial crystallization temperature and crystallinity of PP are reduced after the addition of PDPP.

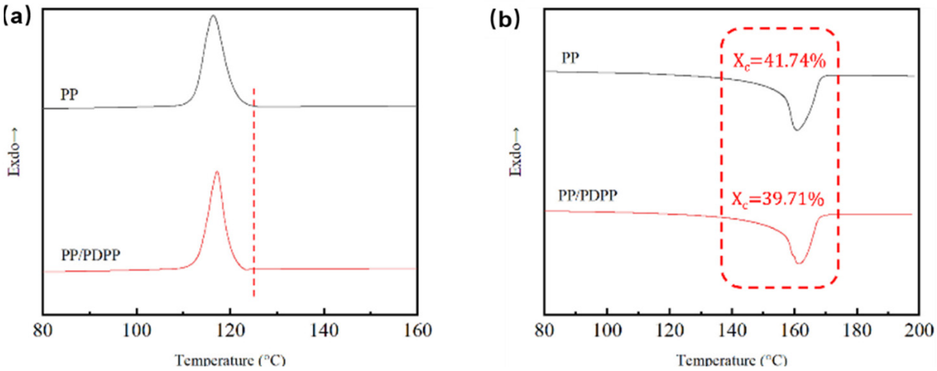

Figure S2. Cooling (a) and melting (b) curves of pure PP and PP/PDPP composite.

4. Effect of PET nanofibrils and PDPP particles on CO_2 adsorption

Figure S3 shows the curve of CO_2 adsorption rates of different samples with time under the conditions of 40℃ and 3000PSI. It can be seen from Figure S3 that when CO_2 is saturated, the adsorption rates of different samples are significantly different, and the CO_2 adsorption rate of PP/PET(F)/PDPP complex is significantly higher than that of PP/PET(F).

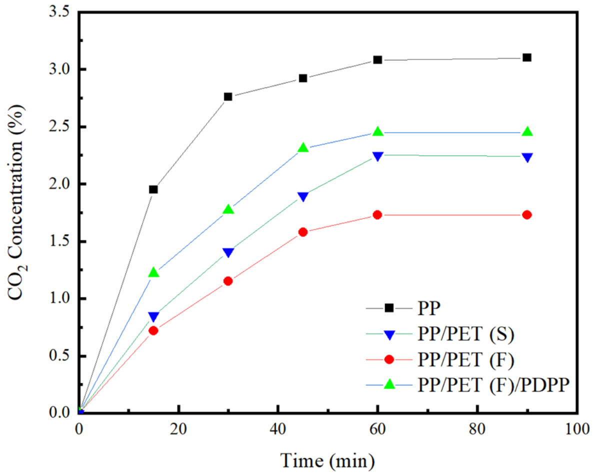

Figure S3. The CO_2 adsorption results as a function of saturation time at 40℃ and 3000Psi.

5. Cellular morphologies of pure PP, PP/PDPP and PP/PET(S) /PDPP foams

As can be seen from Figure S4, the bubble structure of PP/PDPP and PP/PET(S)/PDPP composite foams has not been effectively improved compared with pure PP.

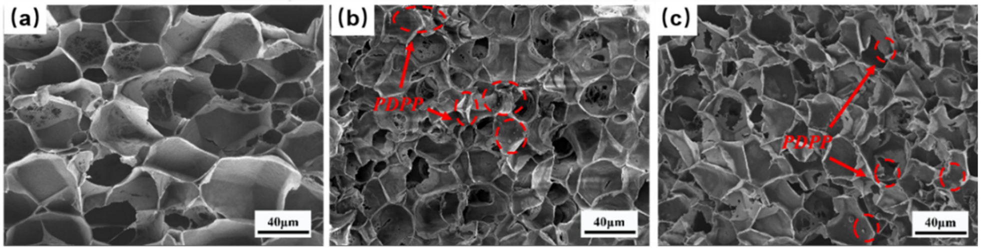

Figure S4. Cellular morphologies of various foams: (a) pure PP, (b) PP/PDPP composite, (c) PP/PET(S)/PDPP composite.

Videos S1-S4. Videos of combustion test for pure PP foam (Video-S1), PP/PET(F) composite foam (Video-S2), PP/PET(S)/PDPP composite foam (Video-S3), and PP/PET(F)/PDPP composite foam (Video-S4)

The corresponding videos are provided in separate files.
